# The Characteristics of Adsorption of Di-n-Butyl Phthalate by Surface Sediment from the Three Gorges Reservoir

**DOI:** 10.3390/toxics12070469

**Published:** 2024-06-27

**Authors:** Yuting Zhang, Min Liu, Li Lin, Liangyuan Zhao, Wei Deng, Cheng Han, Mingli Wu

**Affiliations:** 1 Basin Water Environmental Research Department, Changjiang River Scientific Research Institute, Wuhan 430010, China; 2 Key Lab of Basin Water Resource and Eco-Environmental Science in Hubei Province, Changjiang River Scientific Research Institute, Wuhan 430010, China; 3 Innovation Team for Basin Water Environmental Protection and Governance of Changjiang Water Resources Commission, Wuhan 430010, China

**Keywords:** Three Gorges Reservoir (TGR), sediment, di-n-butyl phthalate (DBP), adsorption characteristics

## Abstract

Phthalic acid esters (PAEs), recognized as endocrine disruptors, are identified as predominant organic pollutants in the Three Gorges Reservoir (TGR). Di-n-butyl phthalate (DBP), a representative PAE, has been extensively studied for its sources, distribution and ecological risks. However, there are few studies on the adsorption of DBP by sediment from the TGR, and the adsorption characteristics of surface sediment on DBP are not clear. Therefore, based on the actual sediment contents and particle sizes in the TGR, the kinetics and isothermal adsorption characteristics of surface sediment on DBP were investigated in this study. The results showed that the equilibrium time was 120 min, the adsorption kinetics were more in line with the pseudo-second-order kinetic model, and the sediment in water from the Yangtze River exhibited a higher adsorption rate and maximum adsorption amount on DBP than that observed in deionized water. Additionally, a decrease in DBP adsorption was observed with increasing sediment content, while sediment particle size and specific surface area had a slight influence. Analysis using SEM, TGA and FTIR revealed that organic matter on the sediment surface significantly contributed to DBP adsorption. This study contributes valuable insights into the adsorption characteristics of DBP by the surface sediment from the TGR, providing a scientific foundation for understanding the migration and transformation of DBP in this critical reservoir in China.

## 1. Introduction

Phthalic acid esters (PAEs) are a group of chemicals used to soften plastics and enhance plasticity and have been widely applied in plastic products, food packaging materials, cosmetics and personal care products [1,2]. It has been reported that PAEs and their metabolites exhibit mutagenic, carcinogenic and teratogenic properties, potentially disrupting human reproductive health, and have been recognized as endocrine-disrupting chemicals [3]. In recent years, due to the extensive industrial production and the widespread use of plastic products, PAEs have continually entered the atmosphere, soil and aquatic environment, posing significant ecological and health risks [4,5]. Consequently, some countries and regions, such as the United States, the European Union, Canada and China, have prioritized the regulation of six specific PAEs as hazardous pollutants [6]. Di-n-butyl phthalate (DBP), a common PAE compound, has been widely detected in major basins in China [2,7,8].

The Three Gorges Reservoir (TGR), a pivotal project for the Yangtze River’s management and development, also functions as a strategic water source in China. It has been reported that the total amount of PAEs in the water and sediment of the main tributaries of the TGR was 4.0~11,697.7 ng/L and 177.3~4744.4 ng/g, respectively, among which DBP was the dominant PAE pollutant [9], indicating that there was a potential ecological risk of DBP in the TGR that should not be ignored. Since the impoundment of the TGR, there has been a significant reduction in the incoming sediment load [10], and the sediment contained in the reservoir ranges generally from 0.02 g/L to 1.5 g/L [11,12]. Moreover, fine particulate sediment with a particle size of less than 0.016 mm accounts for 60.7% of the total sedimentation in the TGR [13]. Pollutants in the water easily adhere to sediment particles, especially on the surface of fine particulate sediment [14]. And the dynamics of sediment transport and pollutant adsorption can significantly alter the distribution and transformation of organic contaminants, thereby influencing the whole aquatic environmental system [15].

Recent studies are mainly focused on the spatiotemporal distribution and ecological risk assessment of DBP in water and sediment [9,16]; however, there are few studies on the adsorption effect of sediment on DBP. It should be noted that the properties of sediment (such as contents and particle sizes) in the TGR are dynamically changed by the influence of different water periods of abundance, flatness and dryness [17]. And the solubility of DBP in water is as low as 11.2 mg/L (25 °C), which is slightly soluble [18]. While the existing literature addresses the adsorption of DBP by sediment, the concentrations examined are typically higher than those found in aquatic environments [19,20]. Therefore, there are few studies based on the actual sediment contents and particle sizes that investigate the adsorption process with a low concentration of DBP, and the adsorption characteristics are still not clear.

This study, considering the sediment contents and particle sizes in the TGR [11,12], as well as the low solubility of DBP in water [18], conducted an investigation on the adsorption characteristics of DBP by surface sediment in the TGR. The microscopic behavior of sediment was analyzed by the SEM, FTIR, TGA and zeta potential characterization. The adsorption kinetics and isotherm of sediment (based on different sediment contents and particle sizes) on DBP were analyzed. Moreover, a comparative study was conducted to discern the differences in the adsorption performance of sediment for DBP between the water from the Yangtze River and the deionized water, which could further reveal the actual adsorption characteristics. This study provides theoretical insights into how sediment in the TGR affects the migration and transformation of DBP, significantly advancing the understanding of DBP dynamics in natural aquatic systems.

## 2. Materials and Methods

### 2.1. Chemicals and Materials

A standard solution of di-n-butyl phthalate (DBP, GR) was purchased from Sigma-Aldrich (St. Louis, MO, USA). Methanol (HPLC) was obtained from Thermo Fisher Scientific (Waltham, MA, USA). And sodium azide (AR) was purchased from Sinopharm Chemical Reagent Co., Ltd. (Shanghai, China).

The surface sediment used in the experiment was collected from Fengjie Basin in the TGR (the background value could be ignored with no DBP being detected). The atomic percentages of surface elements were C—15.29%, O—61.68%, Si—13.35%, Al—4.87%, Fe—1.34%, K—1.13%, Ca—1.09%, Mg—0.85%, Na—0.35% and Mn—0.04%, respectively. The sediment had a large pore diameter with a specific surface area of 8.25 m^2^/g. After the impurities were removed, the sediment was dried and ground to pass through a 100-mesh sieve and then stored for use.

### 2.2. Characterization of Surface Sediment

The morphology of the surface sediment was observed using a ZEISS GeminiSEM 300 field-emission scanning electron microscope (Carl Zeiss AG, Oberkochen, Germany) with a resolution of 1 μm and an acceleration voltage of 15 kV. The thermogravimetric analysis (TGA) was conducted using STA200 equipment (Hitachi, Tokyo, Japan) with alumina (Al_2_O_3_) crucibles in the range from room temperature to 1000 °C with heating ratios of 10 °C/min. The sediment before and after DBP adsorption was measured with FTIR at 4000 to 400 cm^−1^ using an IRTracer 100 infrared spectrophotometer (Shimadzu, Kyoto, Japan). And the zeta potential was measured by a Zetasizer Nano ZS90 zeta potential analyzer (Malvern Panalytical Ltd., Malvern, UK) using water as the dispersant with a pH of 7.49. The specific surface area of the sediment was measured by the Brunauer–Emmett–Teller (BET) method using a 3 Flex adsorption analyzer (Micromeritics Instrument Corporation, Norcross, GA, USA).

### 2.3. Adsorption Experiments

#### 2.3.1. Adsorption Kinetics

A total of 0.05 g of sediment was placed in a 250 mL conical flask, and then 100 mL of 2 mg/L DBP solution was added. Additionally, NaN_3_ was added to inhibit microbial activity. Then, the conical flask was wrapped with a black film, placed in an oscillating incubator at a temperature of 25 °C and shaken at 180 r/min for 0 min, 5 min, 10 min, 20 min, 40 min, 80 min, 2 h, 4 h, 8 h and 12 h, respectively; finally, the concentration of DBP in the supernatant was measured. A control group without adding sediment was also established. The experimental procedure for the adsorption kinetics of DBP in the water from the Yangtze River was the same as described above, with the exception of using river water in place of deionized water. The water from the Yangtze River consisted of 1.84 mg/L TN and 0.05 mg/L TP, and no DBP was detected. The pseudo-first-order kinetic model (Equation (1)) and pseudo-second-order kinetic model (Equation (2)) used in this study can be expressed as follows [21]:

(1) $$Q_{t}=Q_{e}(1-\exp(-k_{1}t))$$

(2) $$Q_{t}=k_{2}Q_{e}^{2}t/(k_{2}Q_{e}t+1)$$

in which *Q_t_* (mg/g) is the adsorption amount at time t, *Q*_e_ (mg/g) is the adsorption amount at equilibrium, *k*_1_ (1/min) is the pseudo-first-order rate constant and *k*_2_ (g/mg·min) is the pseudo-second-order rate constant.

#### 2.3.2. Adsorption Isotherm

A total of 0.05 g of sediment was placed in a 250 mL conical flask, and 100 mL of different concentrations of DBP solution (0.5 mg/L, 1 mg/L, 2 mg/L and 3 mg/L) was added. Then, NaN_3_ was added to inhibit microbial activity, and the conical flask was wrapped with a black film and placed in an oscillating incubator at a temperature of 25 °C with 180 r/min for 2 h; then, the concentration of DBP in the supernatant was measured. Freundlich’s isotherm model (Equation (3)) was used to investigate the isothermal adsorption characteristics of sediment for DBP in this study [22]:

(3) $$Q_{e}=K_{f}{C_{e}}^{\frac{1}{n}}$$

in which *Q*_e_ (mg/g) is the adsorption amount at equilibrium, *C*_e_ (mg/L) is the concentration of DBP at equilibrium, *K_f_* (mg/g)/(mg/L)^n^ is the Freundlich constant and n is an indicator of heterogeneity.

#### 2.3.3. Influence of Sediment Characteristics on Adsorption Isotherm

##### Sediment Content

The sediment content in the TGR is generally 0.02 g/L~1.5 g/L [11,12]. Therefore, according to the experimental procedure in Section 2.3.2, the sediment content was set as 0.5 g/L, 1.0 g/L, 1.5 g/L, 2.0 g/L and 4.0 g/L, respectively, to study the isothermal adsorption of different sediment contents on DBP.

##### Sediment Particle Size

According to the experimental procedure in Section 2.3.2, the sediment particle sizes were set as 60–100 mesh (0.15 mm–0.25 mm), 100–200 mesh (0.075 mm–0.15 mm), 200–320 mesh (0.045 mm–0.075 mm) and more than 320 mesh (<0.045 mm), respectively, to study the isothermal adsorption of different sediment particle sizes on DBP.

### 2.4. Determination of DBP

The experimental suspensions were centrifuged at 4 °C for 10 min at 5000 rpm in a refrigerated centrifuge (Mikro220R, Hettich, Schwarzwald, Germany). Then, the supernatant was taken for the determination of DBP. The concentration of DBP was determined by high-performance liquid chromatography (e2695, Waters, MA, USA) with a detection limit of 0.038 mg/L. The specific determination conditions were as follows: the chromatographic column was a Waters Symmetry column (C18, 4.6 × 250 mm, 5 μm) with a mobile phase of methanol and water (80:20, *v*/*v*) at 35 °C, the injection volume was 10 μL, the flow rate was 1 mL/min and the UV detector wavelength was 220 nm [23,24].

## 3. Results and Discussion

### 3.1. Characterization of Sediment

#### 3.1.1. SEM Analysis

**Figure 1:**
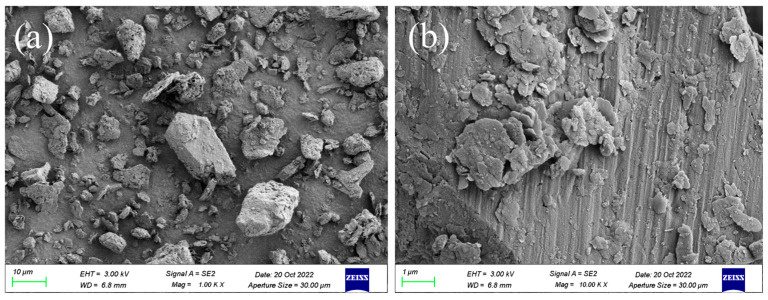
SEM images of sediment: (**a**) 1000× magnification; (**b**) 10,000× magnification, ZEISS, Oberkochen, Germany.

The morphological characteristics of sediment samples are shown in Figure 1. It can be seen that the sizes of sediment particles are not uniform in Figure 1a. However, after further magnification, there are relatively flat areas on the surface of sediment shown in Figure 1b, which might be formed by mineral joints, and there are also some irregular microagglomerations attached to the sediment. These might be caused by biological growth activities, such as extracellular substances secreted by bacteria colonization, growth and proliferation [25], and the other substances, such as humus, existing on the surface of sediment [26] indicate that DBP easily adsorbed to the surface of the sediment.

#### 3.1.2. TGA Analysis

**Figure 2:**
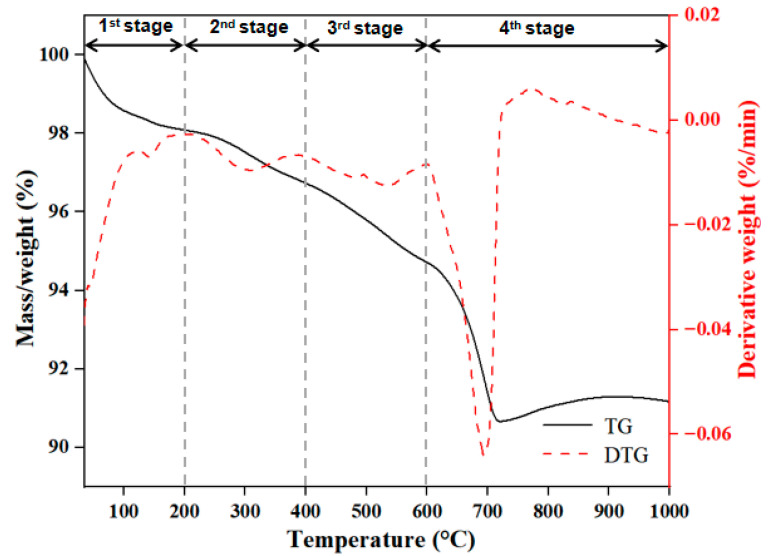
TG and DTG curves during thermal degradation of sediment.

The thermogravimetric (TG) and differential thermogravimetric (DTG) curves during thermal degradation of the sediment are shown in Figure 2. The weight loss of the sample could be divided into four stages, which were 100–200 °C, 200–400 °C, 400–600 °C and 600–1000 °C. At stage 1, there was a 1.3% weight loss at 145 °C, stage 2 exhibited a 1.4% weight loss at 308 °C, stage 3 exhibited a 2.1% weight loss at 536 °C and stage 4 exhibited a 3.6% weight loss at 693 °C. According to the reported study [27], the weight loss in the first stage might be due to the chemically bound water and hygroscopic water of salts and organic matter, the weight loss in the second stage might be associated with decarboxylation and oxidation of organic matter, the weight loss in the third stage might be due to the combustion of different and more recalcitrant classes of organic matter and the weight loss in the fourth stage might be due to the greatest number of carbonate-associated inorganic compounds being degraded during this phase. The results indicated that the sediment from the TGR was composed of organic matter, which could play an important role in the adsorption process of DBP.

#### 3.1.3. FTIR Analysis

**Figure 3:**
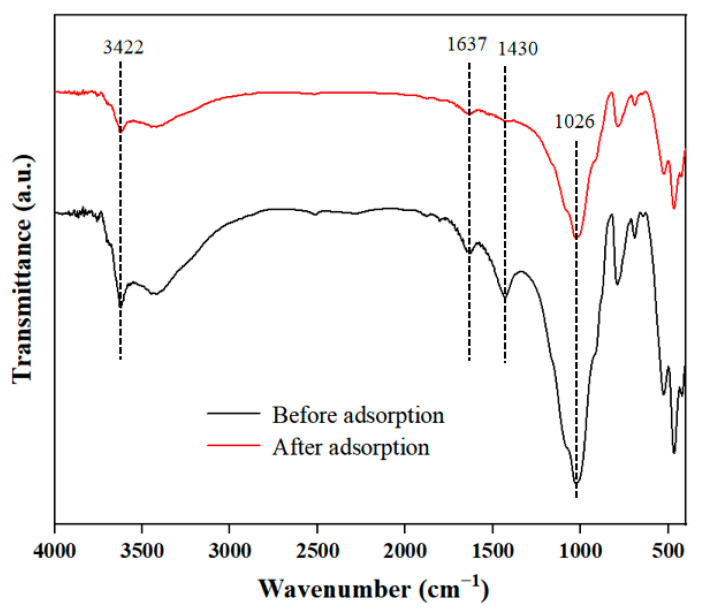
The FTIR results of sediment before and after absorption.

In order to explore the interactions between DBP and sediment, FTIR spectra of sediment before and after adsorption were examined (Figure 3). The peak at 3422 cm^−1^ was attributed to the stretching of the O-H bond of phenolic groups and the N-H bond of amino groups [28]. The peak at 1637 cm^−1^ could originate from the intramolecular and intermolecular hydrogen bond interactions of carboxylic acids, aromatic C=C and C=O in amides [29]. The peak at 1430 cm^−1^ was related to O-H in-plane bending from polysaccharides [30]. The peak at 1026 cm^−1^ mainly resulted from an overlap of the vibrational coupling of C-O stretching [31]. Therefore, the sediment might contain organic matter like carbohydrates and some aromatic structure compounds, which was consistent with the TGA results (Figure 2) and also agreed with the main components of sediment identified in other published research [26]. The difference in peak transmittance before and after adsorption also indicated that these substances had interactions with DBP.

#### 3.1.4. Zeta Potential Analysis

**Figure 4:**
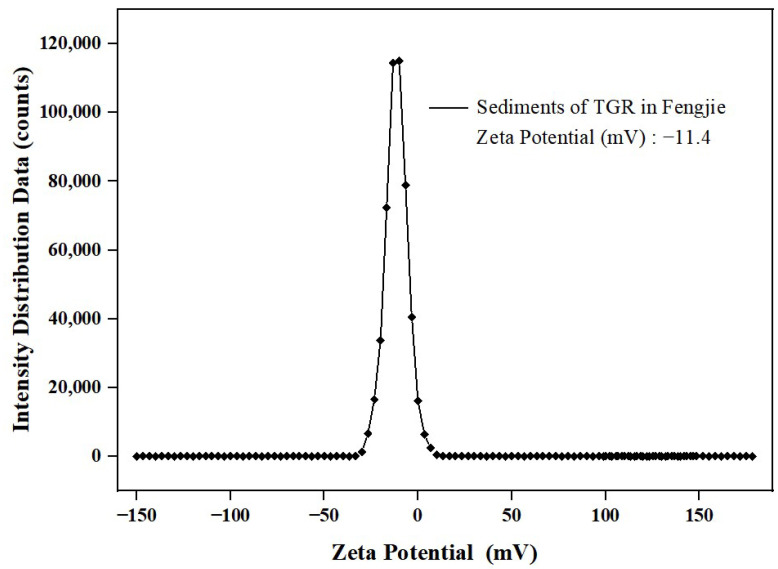
Zeta potential of the sediment.

The zeta potential of sediment commonly exhibits a negative charge [32]. It has been reported that the absolute zeta potential of sediment can represent its stability in water [33]. Generally, a higher absolute zeta potential is associated with greater sediment stability, reducing the likelihood of flocculation [34]. Moreover, elevated zeta potential values reduce sediment collision, leading to an augmented number of adsorption sites and subsequently increasing the adsorption capacity for pollutants [33]. Therefore, the absolute zeta potential can reflect the adsorption capacity of sediment to pollutants to a certain extent. In this study, the zeta potential of sediment is illustrated in Figure 4 as −11.4 mV, which is consistent with the results reported in other studies ranging from −8.53 mV to −18.25 mV [32]. And when the absolute zeta potential surpasses 10 mV, the dispersion system formed by sediment particles in water will disperse from a condensed state; consequently, the adsorption of pollutants can occur easily [35].

### 3.2. Adsorption Kinetics

**Figure 5:**
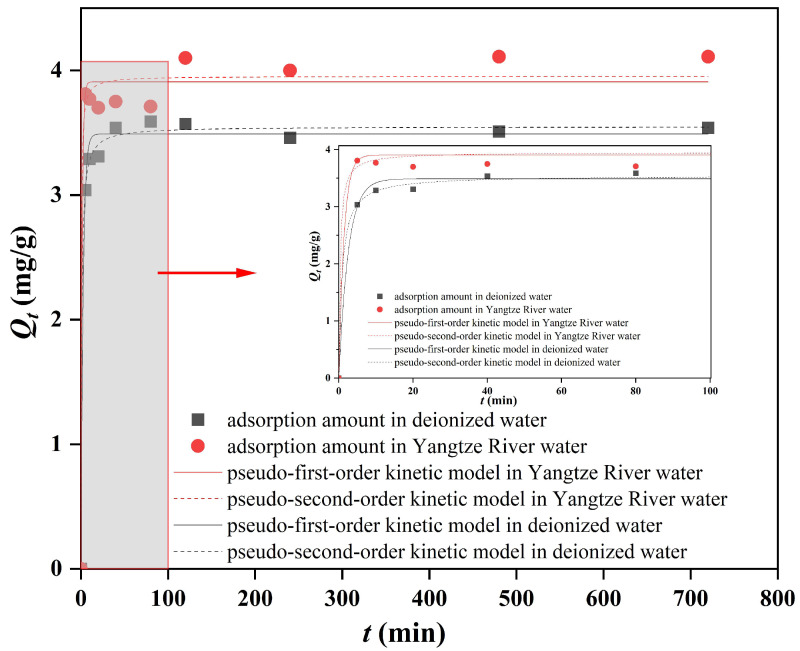
Adsorption kinetics diagrams of DBP by sediment in the deionized water and Yangtze River water.

The adsorption kinetics of sediment on DBP in the deionized water and Yangtze River water are shown in Figure 5. It can be seen that the adsorption amount of sediment on DBP in the deionized water increased rapidly within the initial 40 min and further increased in 40~120 min, while in 120~240 min, the adsorption amount of DBP was in dynamic equilibrium. The adsorption amount of DBP in the Yangtze River water increased rapidly in 5~20 min, remained stable in 20~80 min, then further increased in 80~120 min, while in 120~720 min, the adsorption of DBP was in dynamic equilibrium. Therefore, equilibrium in the adsorption process of the deionized water and Yangtze River water could be achieved after 120 min. This result is consistent with the findings reported in other studies on sediment adsorption of PAE pollutants such as dimethyl phthalate (DMP), di-(2-ethylhexyl) phthalate (DEHP) and dioctyl phthalate (DOP) [36]. It is also worth noting that the adsorption amount of sediment in the Yangtze River water on DBP was higher than that in the deionized water, and its adsorption rate was even faster.

The results of parameters of adsorption kinetics in the deionized water and Yangtze River water are shown in Table 1. The correlation coefficients R^2^ in the pseudo-first-order kinetic model and pseudo-second-order kinetic model in these two adsorption systems are 0.9924 and 0.9796, and 0.9970 and 0.9832, respectively, indicating that the adsorption kinetics of DBP by sediment fit well with both kinds of kinetic curves, while the R^2^ in the latter is a little higher than that of the former—perhaps it is more fitted with the pseudo-second-order kinetic model. In general, the pseudo-second-order kinetic model suggests that the adsorption process involves some chemical changes, such as the formation of chemical bonds between the adsorbent and adsorbate through electron sharing and exchange [37]. Therefore, the adsorption of sediment on DBP in the TGR involved not only a simple diffusive adsorption process but also a chemical adsorption mechanism.

In the pseudo-second-order kinetic model, the adsorption rate *k* and equilibrium adsorption amount *Q*_e_ of DBP in the water from the Yangtze River were 0.746 (g/mg·min) and 3.95 mg/g, respectively, while they were 0.334 (g/mg·min) and 3.55 mg/g, respectively, in the deionized water, as shown in Table 1; therefore, the *k* and *Q*_e_ in the water from the Yangtze River were 2.2 times and 1.1 times that in the deionized water, indicating that the sediment in the water from the Yangtze River had a faster adsorption rate of DBP, which might be attributed to the more complex environment in the water resulting from the actual aquatic system [38]. However, the equilibrium adsorption amount of DBP in the Yangtze water and the deionized water was similar, indicating that the water environment might have little influence on the adsorption capacity of DBP by sediment.

### 3.3. Adsorption Isotherm

#### 3.3.1. Adsorption Isotherm by Different Contents of Sediment

**Figure 6:**
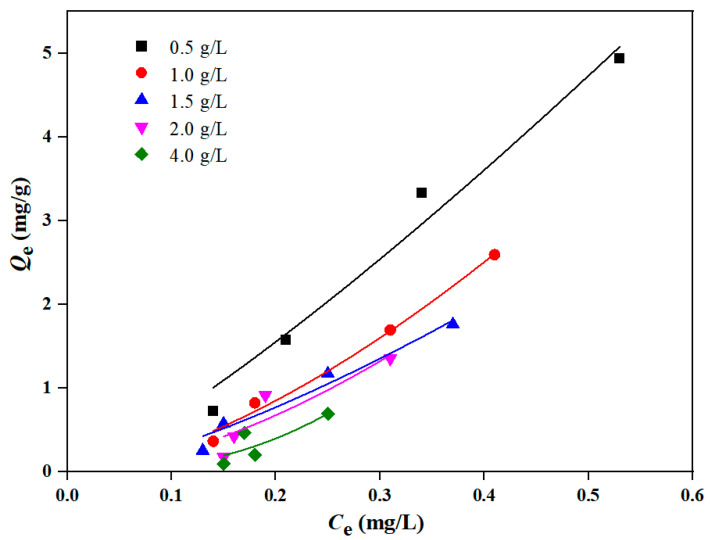
Freundlich isothermal adsorption curve of DBP by different sediment contents.

The Freundlich isotherm model was used to describe the isothermal adsorption experimental results of DBP with different sediment contents (0.5 g/L, 1 g/L, 1.5 g/L, 2 g/L and 4 g/L, respectively), and the results are shown in Figure 6 and Table 2. As shown in Figure 6, the adsorption equilibrium concentration of sediment on DBP gradually decreased with the increase in sediment contents. In addition, with the increase in sediment contents, the adsorption equilibrium amount (*Q*_e_) of DBP gradually decreased. When the sediment content was 0.5 g/L, the adsorption amount of DBP reached its highest value.

Table 2 shows the parameters of the Freundlich isotherm model fitted by the different sediment contents’ adsorption of DBP. When the sediment contents were 0.5 g/L, 1 g/L and 1.5 g/L, the fitted results of the correlation coefficient R^2^ exceeded 0.95; however, when the sediment contents were 2 g/L and 4 g/L, the correlation coefficient R^2^ dropped below 0.85, indicating that with the increase in sediment contents, the adsorption isotherm deviated gradually from the Freundlich model. This might be attributed to the increased content of components, such as humic substances, exerting a significant impact on the adsorption of DBP by sediment [26]. Additionally, it can be seen from Table 2 that when the sediment contents were 0.5 g/L, 1 g/L and 1.5 g/L, the Freundlich constant *K_f_* (mg/g)/(mg/L)^n^ and Freundlich index n gradually decreased, showing a diminishing adsorption performance of sediment on DBP with the increasing sediment contents [39], and it also proved that when the sediment content was 0.5 g/L, the adsorption amount of sediment on DBP reached the maximum.

#### 3.3.2. Adsorption Isotherm by Different Particle Sizes of Sediment

**Figure 7:**
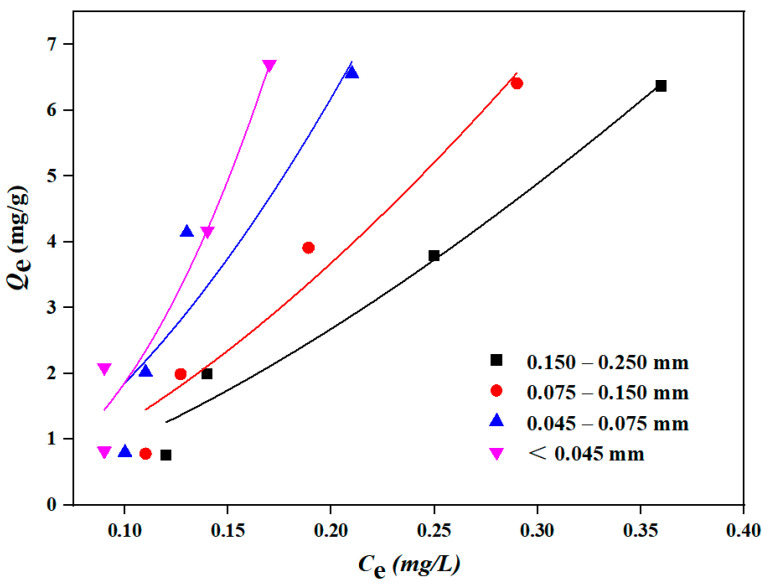
Freundlich isothermal adsorption curve of DBP by sediment with different particle sizes.

The Freundlich model was used to describe the isothermal adsorption experimental results of DBP by sediment with different particle sizes (0.150–0.250 mm, 0.075–0.150 mm, 0.045–0.075 mm and less than 0.045 mm, respectively). The results are shown in Figure 7 and Table 3. As shown in Figure 7, the adsorption equilibrium concentration of DBP gradually decreased with the decrease in sediment particle sizes, and the sediment particle size less than 0.045 mm had the lowest adsorption equilibrium concentration of DBP. However, the adsorption amounts of sediment with different particle sizes on DBP were almost the same, which might be attributed to the prevalence of coarse-grained sediment (particle size larger than 0.025 mm) in this study, where the changes in adsorption amounts for pollutants were less pronounced compared to fine-grained sediment (particle size smaller than 0.025 mm) [40].

Table 3 visually shows the isothermal adsorption performance of different particle sizes of sediment on DBP. The fitted correlation coefficient R^2^ of the Freundlich adsorption isotherm model for DBP by sediment of different particle sizes was always more than 0.85. Additionally, with a decrease in sediment particle size, the specific surface area of each sediment gradually increased, and the Freundlich constant *K_f_* (mg/g)/(mg/L)^n^ also consistently increased, indicating that the adsorption performance of sediment on DBP was continuously enhanced. And it is worth noting that the fine-grained sediment with a particle size less than 0.016 mm constituted 60.7% of the total sediment accumulation in the TGR [13], and it has been reported that the finer the particle size of the sediment, the stronger its adsorption capacity for pollutants, indicating that the sediment in the TGR might have a strong adsorption capacity for DBP.

### 3.4. Comparison of Adsorption Characteristics of DBP by Sediment from the TGR with Other Reports

As there are few reported studies on DBP adsorption by sediment in the TGR, the comparison of adsorption of DBP by soil or sediment in other river basins is shown in Table 4. It can be found that the adsorption of DBP by all adsorbents conforms to the Freundlich model, and the adsorption equilibrium time is mostly 2 h or 3 h, which is consistent with this study (Figure 5). The *K_f_* (mg/g)/(mg/L)^n^ of the studies on the sediment from Zhanjiang Estuary mangrove wetland [20], black soil from Harbin [31] and this study were also gradually increased with the increase in soil or sediment addition, which was consistent with the results in Table 2. Comparing the results of paddy soil from Guangzhou [26] with this study, it can also be found that the larger the specific surface area of the sediment, the larger the *K_f_* (mg/g)/(mg/L)^n^, which is consistent with the results of Table 3. In general, the adsorption characteristics of sediment on DBP are reflected in that the adsorption equilibrium time is about 2 h and the additional amount of sediment and specific surface area have a great influence on the adsorption capacity.

## 4. Conclusions

In this study, surface sediment from Fengjie Basin in the TGR was taken as a representative to investigate the adsorption characteristics on DBP. Analysis by SEM, TAG and FTIR revealed that the organic matter on the surface of sediment played an important role in the adsorption process. The results of zeta potential measurements indicated that the dispersion system formed by sediment particles kept a condensed state, and the adsorption of DBP occurred easily. The adsorption kinetics analysis showed that the adsorption equilibrium time of sediment on DBP was 120 min, and the adsorption process was more fitted with the pseudo-second-order kinetics. The adsorption kinetics differences between the deionized water and Yangtze River water indicated that the adsorption rate (*k*) of sediment to DBP in the water from the Yangtze River was faster but the equilibrium adsorption amounts (*Q*_e_) were very similar, suggesting that the adsorption capacity of sediment on DBP was relatively unaffected by the water environment. The adsorption isotherm showed that the adsorption of DBP by sediment would decrease with an increase in sediment contents; however, the change in sediment particle sizes and specific surface areas would enhance the adsorption capacity but have little effect on the adsorption amount of DBP. In general, this study can provide a scientific basis for understanding the impact of sediment on the migration and transformation of DBP in the TGR, and the adsorption process and mechanism in the actual aquatic environment need further investigation.

## Figures and Tables

**Table 1:** The parameters of adsorption kinetics.

Adsorption System	Kinetics Model	Correlation Coefficients (R^2^)	Rate Constant (*k*_1_, *k*_2_*,* g/mg·min)	Equilibrium Adsorption Amount (*Q*_e_*,* mg/g)
Deionized water	pseudo-first-order	0.9924	0.393	3.49
	pseudo-second-order	0.9970	0.334	3.55
Yangtze River water	pseudo-first-order	0.9796	0.724	3.91
	pseudo-second-order	0.9832	0.746	3.95

**Table 2:** The parameters of Freundlich isothermal adsorption model of DBP by different sediment contents.

Sediment Contents (g/L)	Parameters		
	*K_f_* (mg/g)/(mg/L)^n^	n	R^2^
0.5	11.01	0.8207	0.9799
1	10.49	0.6395	0.9921
1.5	7.199	0.7192	0.9675
2	9.708	0.6027	0.8373
4	22.85	0.3964	0.7510

**Table 3:** The parameters of Freundlich isothermal adsorption model of DBP by different sediment particle sizes.

Sieve (mesh)	Particle Sizes (mm)	Specific Surface Area	Parameters		
		(m^2^/g)	*K_f_* (mg/g)/(mg/L)^n^	n	R^2^
60–100	0.150–0.250	5.38	29.19	0.6738	0.9781
100–200	0.075–0.150	8.29	45.38	0.6406	0.9617
200–320	0.045–0.075	8.96	101.43	0.5756	0.8757
>320	<0.045	9.70	477.98	0.4151	0.9633

**Table 4:** The comparison of adsorption characteristics of different sediment on DBP.

Adsorbents	Particle Sizes (mm)	Specific Surface Area (m^2^/g)	Adsorption Condition	Adsorption Equilibrium Time	Freundlich Model	
					*K_f_* (mg/g)/(mg/L)^n^	n
Sediment from Zhanjiang Estuary mangrove wetland	2.00	NA ^a^	1 g/L sediment, 0.1~8 mg/L DBP	2 h	59.8	1.04
Sediment from Ah-Kung-Dian River	2.00	NA ^a^	200 g/L sediment, 0~2 mg/L DBP	3 h	1.45	1.60
Paddy soil from Guangzhou	0.135	3.90	20 g/L soil, 1~12 mg/L DBP	24 h	0.05	0.73
Black soil from Harbin	2.00	NA ^a^	2.5 g/L soil, 0.5–8 mg/L DBP	2 h	66.32	1.27
Sediment from TGR (this study)	0.150	8.25	0.5 g/L sediment, 0.5~3 mg/L DBP	2 h	11.01	0.82

^a^ Not available.

## Data Availability

Data will be given upon request.
